# Associations of reallocating time between movement behaviours with adiposity and physical fitness among employees: a compositional data analysis

**DOI:** 10.1186/s12889-025-23165-6

**Published:** 2025-05-20

**Authors:** Nucharapon Liangruenrom, Dorothea Dumuid, Zeljko Pedisic, Dyah Anantalia Widyastari, Waris Wongpipit, Piyawat Katewongsa

**Affiliations:** 1 Institute for Population and Social Research, Mahidol University, Phutthamonthon, Nakhon Pathom, Thailand; 2 Allied Health & Human Performance, Alliance for Research in Exercise, Nutrition and Activity, University of South Australia, Adelaide, Australia; 3 Centre for Adolescent Health, Murdoch Children’s Research Institute, Parkville, VIC Australia; 4 School of Public Health, Li Ka Shing Faculty of Medicine, The University of Hong Kong, Hong Kong, China; 5 Faculty of Education, Chulalongkorn University, Bangkok, Thailand; 6 Department of Sports Science and Physical Education, Faculty of Education, Chinese University of Hong Kong, Hong Kong, China

**Keywords:** Isotemporal substitution, Physical inactivity, Sitting, Thailand, Time use, Working adults

## Abstract

**Background:**

Adiposity and low physical fitness are critical public health issues, particularly when taking into consideration the worldwide shift from active to sedentary lifestyles. Thus, the aim of this study was to determine how reallocations of time between sleep, sedentary behaviour (SB), light physical activity (LPA), and moderate-to-vigorous physical activity (MVPA) are associated with adiposity and physical fitness among Thai urban employees.

**Methods:**

Cross-sectional data were collected from a random sample of 424 adults working in Bangkok. Daily durations of SB, LPA, and MVPA were estimated using accelerometers, while sleep duration was obtained from sleep logs. We used body mass index (BMI), percent body fat, and waist circumference as adiposity indicators and maximum oxygen consumption (VO_2 max_), dynamometer-measured handgrip, back and leg strength, and flexibility assessed using sit-and-reach test as fitness indicators.

**Results:**

Reallocating 15 min/day to SB from the remaining behaviours was associated with on average 0.19 mL/kg/min (95% confidence interval [CI]: –0.35, –0.03) lower VO_2 max_. Reallocating 15 min/day to LPA from the remaining behaviours was associated with on average 0.15 kg/m^2^ (95% CI: 0.03, 0.27) higher BMI and 0.34 cm (95% CI: 0.01, 0.67) greater waist circumference. Reallocating 15 min/day to MVPA from the remaining behaviours was associated with on average 1.52 cm (95% CI: –2.85, –0.19) smaller waist circumference and 1.77 cm (95% CI: 0.69, 2.85) greater flexibility.

**Conclusions:**

There is a beneficial association of reallocating more time to MVPA with adiposity and fitness, and a detrimental association of reallocating more time to SB and LPA with adiposity.

**Supplementary Information:**

The online version contains supplementary material available at 10.1186/s12889-025-23165-6.

## Background

Obesity is emerging as one of the primary causes of non-communicable diseases (NCDs) and premature death in the Thai population [1]. According to the National Health Examination Survey (NHES), the prevalence of obesity (defined according to Asia–Pacific perspective as having a body mass index (BMI) of 25 kg/m^2^ or higher) among Thai adults aged 15 years and over, has risen rapidly; from 28.3% in 1997 to 42.4% in 2020 [2, 3]. This alarming trend underscores the growing public health challenge posed by obesity in Thailand. Work commitments may influence lifestyle and shape movement behaviours, including sleep and occupational and non-occupation physical activity (PA) and sedentary behaviour (SB). The fact that obesity rates vary by occupation indicates that occupational activities may play an important role in the genesis of obesity [4, 5]. For example, occupations that involve long sitting or low levels of PA were found to be associated with a higher risk of obesity [6].

Physical fitness is one of key indicators of health in adults, with its specific components such as aerobic capacity and muscular strength being strongly associated with the risk of various NCDs [7]. The importance of maintaining adequate levels of physical fitness is highlighted in the World Health Organization’s (WHO) PA guidelines, which encourage adults to regularly engage in aerobic and muscle-strengthening activity to improve health and maintain physical fitness [8]. However, a recent international study across six European countries found that the level of physical fitness among employees is low, particularly muscular endurance among females [9].

Adiposity and low physical fitness are critical public health issues, particularly when taking into consideration the worldwide shift from active to sedentary lifestyles, and Thailand is no exception to this trend [10]. In particular, employed adults in Thailand are becoming increasingly sedentary, often at the expense of their PA [11]. This trend is particularly pronounced in urban areas, where the sedentary nature of many jobs, coupled with the widespread “unhealthy” changes to lifestyle, exacerbates the problem of obesity [2] and potentially poor physical fitness.

Systematic reviews suggest that movement behaviours, including PA, SB, and sleep are each individually associated with adiposity indicators, such as BMI, body fat percentage, and waist circumference, as well as with cardiorespiratory and physical fitness among adults [12–16]. However, times spent in these movement behaviours are portions of the 24-h day, and therefore, they are co-dependent and produce combined effects on health [17–19]. Thus, an integrated approach to understanding the distribution of time spent in movement behaviours and how this relates to different health indicators has been recommended [17].

Compositional data analysis (CoDA) can be used to explore relationships of movement behaviours with health, while adequately addressing specific mathematical properties of time use compositions [17, 18]. However, CoDA has rarely been used in the context of low- and middle-income countries. In specific, a recent review of 103 CoDA-based studies on health outcomes associated with reallocations of time found that less than 10% of included studies were from low- and middle-income countries [20].

The review also found that reallocating time to SB from light physical activity (LPA) or sleep is unfavourably associated with adiposity and physical fitness, and vice versa [20]. The strongest benefits were suggested for reallocations of time to moderate-to-vigorous physical activity (MVPA) from any of the other movement behaviours [18, 20]. It is therefore very plausible that the rising prevalence of obesity and poor physical fitness among Thai employees is closely linked to the recent shifts in their distributions of time spent in movement behaviours.

However, the combined associations of movement behaviours with adiposity and physical fitness among Thai employees have not been empirically examined. Two previous Thai studies analysed PA, SB, and sleep integratively [11, 21], but they were focused on examining prevalence, trends, and correlates of these behaviours.

Thus, the aim of this study was to determine how reallocations of time between PA, SB, and sleep are associated with indicators of adiposity and physical fitness among Thai urban employees using CoDA.

## Methods

### Study design and participants

In this cross-sectional study conducted between September 2022 and June 2023, participants were recruited from the population of working adults aged 18–59 years. A simple random sampling was carried out to select workplaces in the Bangkok metropolitian area from the database of Thai Ministry of Commerce. Seven out of 20 randomly selected workplaces accepted the invitation to join the study and allowed us to invite their employees to participate. Their adult employees with full-time employment were invited to participate, if they could read, write and communicate fluently in Thai, were not pregnant at the time of the survey, and received a passing score from the Physical Activity Readiness Questionnaire (PAR-Q +). We used the Thai version of PAR-Q + to assess severity of major cardiovascular events and musculoskeletal injuries to determine eligibility of employees to participate in the remaining parts of the study [22]. This was done to ensure participants’ readiness for physical fitness tests. A total of 424 working adults participated in the study, but after excluding 65 participants with invalid accelerometer data, the final analytical sample included 359 individuals.

### Measurement of movement behaviours

The PA and SB data were collected using the Actigraph GT9X Link accelerometer (ActiGraph, LLC., Pensacola, FL, USA). Participants were asked to wear the device attached on an elastic waist band at their right hip for at least seven consecutive days during waking hours. Participants would remove the device at bedtime and for bathing or water-based activities. They also recorded time and reasons for not wearing the accelerometer (e.g. sleep, swimming) in an activity log. Accelerometer data were processed using the version 6.13.4 of ActiLife software (ActiGraph, LLC., Pensacola, FL, USA). A sampling rate of 50 Hz with 60-s epoches was used. A non-wear period was defined as ≥ 90 consecutive minutes of zero counts with the allowance of up to 2-min intervals of < 100 counts per minute [23]. Accelerometry days with wear time of ≥ 10 h/day were considered valid. Participants with ≥ 4 days of valid accelerometer data and with at least one valid weekend day were included in the analysis. The cutoffs by Troiano et al. [24] were used to classify each 1-min epoch as SB (< 100 counts per minute), LPA (100–2019 counts per minute), or MVPA (≥ 2020 counts per minute) [24]. The average daily wear time was 845.45 min (standard deviation = 69.54). Minutes of SB, LPA, and MVPA were averaged across all valid days using a 5: 2 weighting for weekdays versus weekend days.

Data on sleep duration were collected over seven days, using sleep logs in which participants recorded the times they went to bed and woke up each day. Sleep duration on each day of data collection was estimated as the amount of time between going to bed and waking up, including night-time and daytime sleep. This measure represents the sleep period time, that among some participants may include *wake after sleep onset* time. The average sleep duration was calculated across all valid days, using the same weights for weekdays and weekend days as for the remaining behaviours. The sum of estimated daily times spent in sleep, SB, LPA and MVPA did not amount to 1440 min for all participants (the average sum across the whole sample was 1,362.81 min). Therefore, the daily amounts of time in sleep, SB, LPA and MVPA were linearly adjusted so that they add up to a total of 24 h.

### Measures of adiposity and physical fitness

Data on adiposity and physical fitness were collected and recorded by exercise science technicians (see Additional file 1 the recording form). The measures of adiposity included BMI, body fat percentage, and waist circumference. BMI was calculated from body weight and height measured using the body composition monitor (BC-587, Tanita Corp, Tokyo, Japan) and stadiometer (Seca 217, Seca, Hamburg, Deutschland), respectively. Percent body fat was measured using the bioelectrical impedance analysis from the same body composition monitor. A tape measure was used to assess waist circumference following WHO protocols. [25] Body height and waist circumference were measured twice, and, if the difference between the first two measures was more than 10 percent, a third measure was taken. The arithmetic mean of all available measurements was used in the analysis.

Five physical fitness tests were performed. Maximum oxygen consumption (VO_2 max_) was estimated using the Queens College Step Test [26]. Participants stepped up and down on a wooden box (41.3 cm high) for three minutes at a rate of 24 steps/minute (with a metronome set at 96 bpm) for males, and at 22 steps/minute (with a metronome set at 88 bpm) for females. The pulse rate was counted by a technician for 15 s at the end of the test. The number of pulses was multiplied by 4 to estimate the heart rate per minute and VO_2 max_ using the following formulas for men: VO_2 max_ (mL/kg/min) = 111.33 – [0.42 × heart rate (bpm)], and for women: VO_2 max_ (mL/kg/min) = 65.81 – [0.1847 × heart rate (bpm)]. Handgrip strength was assessed by the handgrip dynamometer (TKK Model 5401; Takei, Tokyo, Japan). Participants were asked to use one hand at a time to hold the handle of the device and squeeze it as hard as they could. They performed this two times for each hand, and the higher of the two scores was used in the analysis. The back and leg dynamometer (TKK Model 5402; Takei, Tokyo, Japan) was used to measure back and leg strength. For both measurements, participants stood upright on the dynamometer and pulled the handle with both hands as hard as they could. For the leg strength measurement specifically, participants bent their knees so that the angle between upper and lower leg was approximately 110 degrees. Two measurements were taken separately for back and leg strength tests, and the higher of the two scores was used in the analysis. The sit-and-reach test was used to assess the flexibility of lumbar back and posterior thigh muscles. Participants sat with their legs stretched out and they were asked to bend forward as much as they can. The most distant point they could reach with their fingertips was measured using a sit-and-reach box and expressed in centimeters. They performed the test twice and the longer distance was used in the analysis.

### Other measures

Face-to-face interviews were conducted to collect information about participant sociodemographic characteristics (see Additional file 2 the questionnaire), including sex (female and male), age, education level (below bachelor degree, bachelor degree, and higher than bachelor degree), marital status (never married and currently or previously married), monthly income (lowest, low, moderate, high, and highest), and predominant posture at work (sitting and non-sitting), and lifestyle characteristics, including smoking (never smoked, former smoker, and current smoker), alcohol consumption (non-drinker, light-to-moderate drinker, and heavy drinker), daily sweetened beverage intake (none, light, moderate, and heavy), and daily fruit and vegetable intake (sufficient and insufficient). Brachial blood pressure was measured using Omron 7156 oscillometric device (Omron Corporation, Kyoto, Japan) after participants took rest for at least 5 min, and categorised as normal (< 140 mm Hg/< 90 mm Hg) or high (≥ 140 mm Hg/≥ 90 mm Hg).

### Data analysis

Descriptive statistics including absolute frequency, percentage, median, and interquartile range (IQR) were used to describe sample characteristics. Differences in adiposity markers and physical fitness by sociodemographic and lifestyle characteristics were tested for statistic significance using one-way univariate analysis of variance (ANOVA). Descriptive statistics for a four-part composition of movement behaviours included the compositional mean and pairwise log-ratio variation matrix. To explore the association between movement behaviour composition and dependent variables (i.e. BMI, body fat percentage, waist circumference, VO_2 max_, handgrip strength, leg strength, back strength, and flexibility), we used a CoDA approach based on multiple linear regression models [19]. First, compositional data were checked for the presence of zero values, and no such values were found. The four compositional parts were expressed as a set of three isometric log ratio (*ilr*) coordinates. A specific type of *ilr* (pivot *ilr*) was used, so that the first coordinate of each set captures the relative dominance of one behaviour (e.g., sleep) over all the remaining behaviours (SB, LPA, and MVPA). Four sets of *ilr* coordinates were created, each time permutating the order of compositional parts, so that each set of *ilrs* captured dominance of a different behaviour relative to all the remaining behaviours in its first coordinate. The four sets of *ilrs* were included as predictors in four sets of regression models, with health indicators as the dependent variables. The beta coefficient of the first *ilr* coordinate from each of these four models was used to determine the relationship between one behaviour (relative to the remaining behaviours) and a given health outcome. Multiple determination coefficient was used to determine the associations between the set of *ilr* coordinates (the overall composition) and the outcomes.

Differences in health outcomes associated with reallocations of time between movement behaviours were estimated using the CoDA regression models from above [27]. For each outcome variable, we calculated an estimated difference associated with theoretical reallocations of 5, 10, and 15 min of time to one behaviour from all other behaviours (i.e., one-for-remaining reallocation), with the sample mean as the reference composition. The absolute differences and corresponding 95% confidence intervals were calculated and plotted.

All regression models were adjusted for sex, age, education level, marital status, monthly income, predominant posture at work, smoking, alcohol consumption, daily sweetened beverage intake, daily fruit and vegetable intake, and blood pressure. Statistical significance was set at a *p* < 0.05. The analyses were conducted using ‘compositions’ [28], ‘dplyr’ [29], ‘car’ [30], and ‘codaredistlm’ [31] packages in R software (R Foundation for Statistical Computing, Vienna, Austria).

## Results

### Sample characteristics

The median age of participants in this study was 36 years (IQR = 14). Most of the participants were females (63.79%), had finished a bachelor degree (73.74%), and worked predominantly in a sitting position (71.79%). Somewhat less than a half of the employees were currently or previously married (44.97%) and had moderate monthly income (41.90%). In terms of health, most of the participants had insufficient daily fruit and vegetable intake (89.42%), had normal blood pressure (85.47%), and never smoked (84.12%). The median BMI was 24.31 (kg/m^2^) representing an overweight status. Median fitness scores of leg (71 kg) and back (61 kg) reflected very low strength of leg and back muscles for males (< 137 kg for leg and < 91 kg for back) and moderate strength (66–113 kg for leg and 52–97 kg for back) for females, according to Corbin et al. [32] (Table 1).

**Table 1:** Characteristics of participants (*n* = 359)

Variable	*n* [%]
**Sex**	
• Female	229 [63.79]
• Male	130 [36.21]
**Highest education level**	
• Below bachelor degree	31 [8.66]
• Bachelor degree	264 [73.74]
• Higher than bachelor degree	63 [17.60]
**Marital status**	
• Never married	197 [55.03]
• Currently or previously married	161 [44.97]
Monthly income*	
• Very low (≤ 10,000 baht)	2 [0.56]
• Low (10,001–20,000 baht)	117 [32.68]
• Moderate (20,001–40,000 baht)	150 [41.90]
• High (40,001–100,000 baht)	81 [22.63]
• Very high (> 100,000 baht)	8 [2.23]
**Predominant posture at work**	
• Sitting	257 [71.79]
• Non-sitting	101 [28.21]
**Smoking**	
• Never smoked	302 [84.12]
• Former smoker	20 [5.57]
• Current smoker	37 [10.31]
**Alcohol consumption**	
• Non-drinker	165 [45.96]
• Light to moderate drinker	170 [47.35]
• Heavy drinker	24 [6.69]
**Daily sweetened beverage intake**	
• None	61 [16.99]
• Light	115 [32.03]
• Moderate	167 [46.52]
• Heavy	16 [4.46]
**Daily fruit and vegetable intake**	
• Sufficient	38 [10.58]
• Insufficient	321 [89.42]
**Blood pressure**	
• Normal	306 [85.47]
• High	52 [14.53]
	**Median [IQR]**
**Age (years)**	36.00 [14.00]
**Adiposity markers**	
• BMI (kg/m^2^)	24.31 [5.89]
• Percent body fat	28.80 [10.90]
• Waist circumference (cm)	81.00 [18.25]
**Physical fitness**	
• VO_2 max_ (mL/kg/min)	37.74 [8.87]
• Handgrip strength (kg)	27.50 [13.30]
• Leg strength (kg)	71.00 [52.88]
• Back strength (kg)	61.00 [41.88]
• Flexibility (cm)	7.00 [13.00]

*BMI* body mass index, *VO*_*2 max*_ maximum oxygen consumption, *IQR* interquartile range

^*^1 United States Dollar = 33.6 Thai Baht (as of January 31, 2025)

Males had higher BMI and waist circumference, while females had higher percent body fat (*p* < 0.001 for all; Additional file 3). Females performed better in the flexibility test, compared with males (*p* = 0.001). In all other physical fitness tests, males had higher scores (*p* < 0.001 for all).

### Sleep, sedentary behaviour, and physical activity

The compositional mean included 515 min/day (35.8%) of sleep, 662 min/day (46.0%) of SB, 251 min/day (17.4%) of LPA, and 12 min/day (0.8%) of MVPA.

Proportionality between the compositional parts for the total sample is presented in Table 2. The highest pairwise log-ratio variance (*t*) was observed for SB and MVPA (*t* = 0.80), indicating that the time spent in SB was least proportional with MVPA. The lowest pairwise log-ratio variance was observed for sleep and SB (*t* = 0.05), indicating that sleep and SB had the highest co-dependence.

**Table 2:** Variation matrix for the time-use composition

Time-use component	Sleep	SB	LPA	MVPA
Sleep	0.00	0.05	0.10	0.76
SB	0.05	0.00	0.12	0.80
LPA	0.10	0.12	0.00	0.71
MVPA	0.76	0.80	0.71	0.00

*SB* sedentary behaviour, *LPA* light physical activity, *MVPA* moderate-to-vigorous physical activity

Values represent pairwise log-ratio variances (*t*) of the respective behaviours

### Relationship of movement behaviours with adiposity and fitness

More time spent in SB, relative to the remaining behaviours, was associated with lower VO_2 max_ (*β* = –5.20, *p* = 0.023; Table 3). Specifically, reallocating 15 min/day to SB from the remaining behaviours was associated with on average 0.19 mL/kg/min (95% confidence interval [CI]: –0.35, –0.03) lower VO_2 max_.

**Table 3:** Relationships of the first pivot coordinate of the time-use composition (independent variable) with adiposity markers and physical fitness (dependent variables): results of a set of multiple linear regression analyses

Movement behaviour	BMI		Body fat percentage		Waist circumference		VO_2 max_		Handgrip strength		Leg strength		Back strength		Flexibility	
	***β***	***p***	***β***	***p***	***β***	***p***	***β***	***p***	***β***	***p***	***β***	***p***	***β***	***p***	***β***	***p***
Sleep vs remaining	–0.90	0.504	–3.50	0.296	–0.89	0.808	2.53	0.285	1.10	0.522	3.07	0.750	–2.82	0.659	–2.05	0.491
SB vs remaining	–0.87	0.507	0.37	0.910	–2.45	0.491	–**5.20**	**0.023**	–0.50	0.760	–2.36	0.802	–4.09	0.512	–1.15	0.690
LPA vs remaining	**2.42**	**0.017**	4.60	0.066	**5.54**	**0.044**	2.65	0.138	–0.50	0.710	1.05	0.884	8.21	0.088	0.65	0.770
MVPA vs remaining	–0.64	0.075	–1.47	0.098	–**2.19**	**0.025**	0.03	0.969	–0.10	0.805	–1.76	0.491	–1.30	0.444	**2.55**	**0.001**

*β* = unstandardised regression coefficient for the first pivot coordinate, that is, an isometric log ratio of the behaviour of interest, relative to the geometric mean of the remaining behaviours

*p p*-value for *β, SB* sedentary behaviour, *LPA* light physical activity, *MVPA* moderate-to-vigorous physical activity, *Remaining* remaining movement behaviours, *BMI* body mass index, *VO*_*2 max*_ maximum oxygen consumption, Significant associations (*p* < 0.050) are highlighted in bold; Each estimate was adjusted for sex, age, education level, marital status, monthly income, predominant posture at work, smoking, alcohol consumption, daily sweetened beverage intake, daily fruit and vegetable intake, and blood pressure

**Fig. 1:**
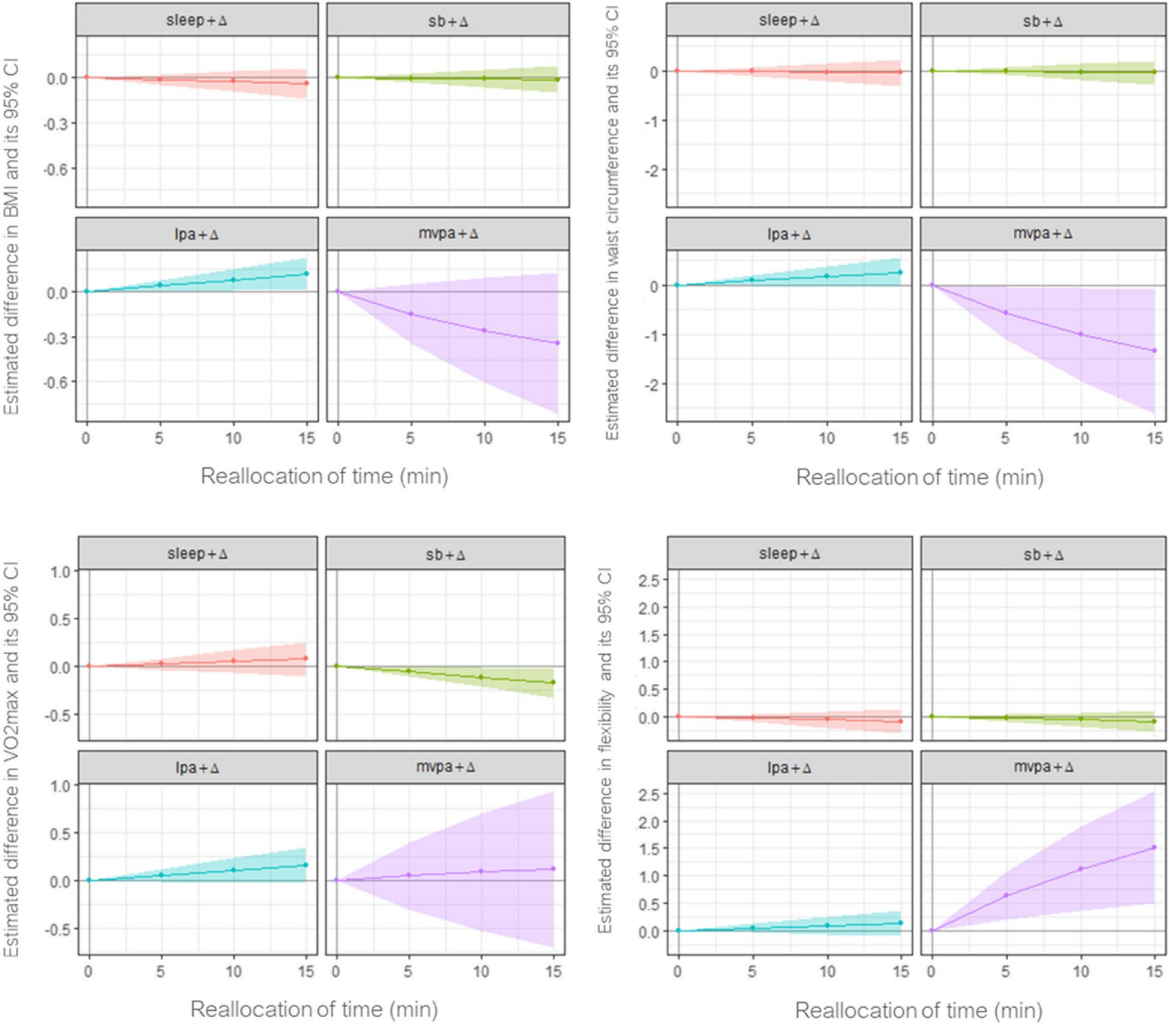
Estimated differences in BMI, waist circumference, VO2 max, and flexibility associated with reallocations of up to 15 mininutes between movement behaviours. Time is added to the behaviour in the header, drawn pro-rata from the remaining behaviours. Notes: Δ = added time; SB = sedentary behaviour; LPA = light physical activity; MVPA = moderate-to-vigorous physical activity; BMI = body mass index; VO2 max = maximum oxygen consumption; CI = confidence interval; Estimates were adjusted for sex, age, education level, marital status, monthly income, predominant posture at work, smoking, alcohol consumption, daily sweetened beverage intake, daily fruit and vegetable intake, and blood pressure

More time spent in LPA, relative to the remaining movement behaviours, was associated with higher BMI (*β* = 2.42, *p* = 0.017) and greater waist circumference (*β* = 5.54,* p* = 0.044). Specifically, reallocating 15 min/day to LPA from the remaining behaviours was associated with on average 0.15 kg/m^2^ (95% CI: 0.03, 0.27) higher BMI and 0.34 cm (95% CI: 0.01, 0.67) greater waist circumference (Fig. 1).

More time spent in MVPA, relative to the remaining behaviours, was associated with smaller waist circumference (*β* = –2.19, *p* = 0.025) and greater flexibility (*β* = 2.55, *p* = 0.001). Specifically, reallocating 15 min/day to MVPA from the remaining behaviours was associated with on average 1.52 cm (95% CI: –2.85, –0.19) smaller waist circumference and 1.77 cm (95% CI: 0.69, 2.85) greater flexibility.

## Discussion

The main finding of this study is that movement behaviours are associated with markers of adiposity and physical fitness among Thai urban employees. Specifically, more SB is detrimentally associated with VO_2 max_, more LPA is detrimentally associated with BMI and waist circumference, and more MVPA is beneficially associated with waist circumference and flexibility.

To interpret these findings within CoDA framework, it is important to clarify that the “reallocation of time” to a given behaviour reflects a hypothetical increase (e.g., 15 min per day) in that behaviour, with the equivalent amount of time taken proportionally from the remaining parts of the time-use composition [27]. For example, the finding that more SB is associated with lower VO_2 max_ suggests that a theoretical increase in SB at the expense of sleep, LPA and MVPA is associated with poorer cardiorespiratory fitness. Given that the times spent in sleep, SB, LPA and MVPA always add up to 24 h per day, one movement behaviour cannnot be indepedently associated with a health outcome. Therefore, all associations presented in this paper should be interpreted by considering the whole time-use composition.

For VO_2 max_, our study revealed a negative association with SB, but we did not find a significant association with MVPA. This is somewhat unexpected, because MVPA is considered a primary driver of improved cardiorespiratory fitness, including VO_2 max_. [33] However, a previous study suggested that only leisure time (but not occupational) MVPA is favourably associated with cardiorespiratory fitness [34, 35]. Another study found a stronger association of leisure-time MVPA. It might be that the participants in our sample, who were all employees, accrued their MVPA mostly at work, which would explain the unexpected result. The unfavourable association between SB and VO_2 max_ is consistent with findings of a recent systematic review, which reported that higher device-measured sedentary time is associated with poorer cardiorespiratory fitness among adults [36]. This was also found in a study that analysed associations of cardiorespiratory fitness with a time-use composition consisting of SB, LPA and MVPA using CoDA [37]. Given that poor cardiorespiratory fitness is associated with a higher risk of all-cause mortality [38], our finding may be particularly important for adults who spend a lot of their time in sedentary behaviour, such as office workers.

Detrimental associations between LPA and adiposity have been reported in previous studies, with an accompanying potential explanation that some of the LPA may have been misclassified and that it actually partially represents SB [18, 39]. It may be that such misclassification occurred in our study as well. Another possible explanation of our findings is that in the theoretical one-for-remaining reallocations, adding time to LPA was done partially at the expense of MVPA; a movement behaviour that was favourably associated with adiposity markers in this and previous studies [20].

Favourable associations of MVPA with waist circumference found in our study are in line with previous evidence [20]. Engaging in MVPA may help create negative energy balance [40] and improve fat oxidation [41], which could explain our finding. Furthermore, the favourable association between MVPA and flexibility is in line with the previous studies which suggested that PA interventions may improve flexibility among employees [42, 43]. Several types of exercise can be used to increase the range of joint motion [44]. It may be that the employees involved in the current study performed such exercises as part or alongside their MVPA, which would explain our finding. A certain level of flexibility is necessary for engagement in some types of PA. Therefore, it may be that employees with lower flexibility were less likely to engage in MVPA, or that the association between MVPA and flexibility is bidirectional.

### Strengths and limitations

The key methodological strengths of this study include: (1) a random sample that was representative of the working population in the metropolitan area of the largest city in Thailand; (2) various indicators of adiposity and fitness that were considered; (3) the use of accelerometers to assess PA and SB; and (4) the use of CoDA to enable the integrated analysis of movement behaviours while adequately acknowledging compositional properties of time-use data.

However, there are also some limitations to this study. First, the use of a cross-sectional study design prevented us from establishing causal relationships. Second, the necessity of conducting all measurements during working hours may have introduced selection bias, where companies with greater resources and more flexibility in scheduling their work were probably more likely to participate. Third, the data were collected during the COVID-19 pandemic; a period in which the movement-behaviour composition may have substantially changed [45, 46]. However, it should be noted that our study was not conducted during the peak of the pandemic. Fourth, sleep duration was obtained from sleep logs. However, the potential recall bias was reduced by asking participants to keep logs on a daily basis. Fifth, in our study, we used the step test, which provides an indirect estimate of VO_2 max_. While the step test is a valid method for assessing VO_2 max_ among generally healthy adults [26], its use might have affected our findings. A direct measurement of VO_2 max_ through the maximal graded exercise test and gas exchange analysis would be preferred, but it is resource-intensive and less feasible in large-scale, epidemiological studies, such as ours. Finally, the way we conceptualised theoretical reallocations of time in this study may not reflect the true nature of reallocations that would occur in the study population. To theoretically increase the amount of time spent in a given behaviour, we assumed that reallocated amounts of time from the remaining behaviours are proportional to geometric means of these behaviours (i.e. more time is taken away from behaviours with higher geometric means). However, “time flows” that are associated with an increase of time spent in one behaviour (i.e. the ways parts of the time-use composition are readjusted) are highly complex and are likely to vary significantly within and between individuals [47]. Hence, we could have conceptualised theoretical reallocations in a different way. However, this was beyond the scope of our study.

### Implications for public health

Our findings provide justification for targeted interventions to reduce SB while increasing MVPA among Thai employees, as such interventions could lead to improved adiposity status and physical fitness in this large and important population group. A range of effective interventions that could serve for this purpose have been identified in previous studies [48, 49]. Such interventions could complement the Thai National Action Plan on Physical Activity 2018–2030 [50], endorsed by the Ministry of Public Health and its partners, which aligns with the Global Action Plan on Physical Activity 2018–2030 [51] to promote the implementation of healthy workplace programs. This would help increase the number of employees in Thailand that meet the Thai national Recommendations for Physical Activity, Non-Sedentary Lifestyles, and Sleep [52].

## Conclusions

Our study shows that how daily time is allocated to movement behaviours has important links with adiposity and fitness among Thai urban employees. There is a beneficial association of reallocating more time to MVPA with adiposity and fitness, and a detrimental association of reallocating more time to SB with adiposity. The observed associations between LPA and higher BMI and waist circumference suggest that LPA alone may not be sufficient to counteract the adverse effects of sedentary lifestyles. These insights are important for developing targeted interventions to address the growing prevalence of obesity and poor physical fitness in Thailand's working population, especially in urban settings.

Future longitudinal and intervention CoDA-based studies are needed, to establish causal relationships of movement behaviour with adiposity and fitness among employees. To measure sleep duration, such studies should consider using accelerometry alongside or instead of sleep logs.

## Supplementary Information

Additional file 1. Adiposity and Physical Fitness Recording Form the English version of the recording form of adiposity and physical fitness tests, including age, blood pressure, body composition, flexibility, strength, and cardiovascular fitness and aerobic edurance.

Additional file 2. Sociodemographic and health behaviour questionnaire the questionnaire translated into English to collect data on participants’ personal information, sociodemographic characteristics, and health behaviours.

Additional file 3. Arithmetic means of adiposity markers and physical fitness by sample characteristics the detailed table of arithmetic means of adiposity markers and physical fitness by sample characteristics.

## Data Availability

The datasets without participants’ confidential data will be available upon a reasonable request with written letter from an authorised organisation.

## Abbreviations

ANOVA: Analysis of Variance
BMI: Body Mass Index
CoDA: Compositional data analysis
CI: Confidence Interval
IQR: Interquartile Range
ILR: Isometric Log Ratio
LPA: Light Physical Activity
VO_2 max_: Maximum Oxygen Consumption
MVPA: Moderate-to-Vigorous Physical Activity
NHES: National Health Examination Survey
NCDs: Non-Communicable Diseases
PA: Physical Activity
PAR-Q +: Physical Activity Readiness Questionnaire
SB: Sedentary Behaviour
WHO: World Health Organization
