# Supplementary material for: Associations of reallocating time between movement behaviours with adiposity and physical fitness among employees: a compositional data analysis
Source: BMC Public Health. 2025 May 20;25:1861. doi: 10.1186/s12889-025-23165-6 (PMC12090509; doi:10.1186/s12889-025-23165-6)
Supplement: Supplementary file 1 — Additional file 1. Adiposity and Physical Fitness Recording Form the English version of the recording form of adiposity and physical fitness tests, including age, blood pressure, body composition, flexibility, strength, and cardiovascular fitness and aerobic edurance. [file 12889_2025_23165_MOESM1_ESM.docx]

**Adiposity and Physical Fitness Recording Form**

**‘Development of Academic Tools and Evidence to Support Policy Design and Decision Making in Physical Activity Promotion to Reduce NCDs’ Project**

Institute for Population and Social Research, Mahidol University

**Respondent ID** 🞎🞎-🞎🞎-🞎🞎-🞎🞎🞎

**(Date – Month – Interviewer no. – Respondent no.)**

Accelerometer no. …………………………………………………

|  | **1^st^ test** | **2^nd^ test** | **3^rd^ test  (if required)** | **Average** |
| --- | --- | --- | --- | --- |
| **Age and Blood pressure** | | | | |
| - Age (years) |  |  |  |  |
| - Blood pressure (mm Hg) | / | / | / | / |
| **Adiposity and Physical Fitness Tests** | | | | |
| **1. Body composition** | | | | |
| - Weight (kg) |  |  |  |  |
| - Height (m) |  |  |  |  |
| - Body mass index (kg/m^2^) |  |  |  |  |
| - Waist circumference (cm) |  |  |  |  |
| - Hip circumference (cm) |  |  |  |  |
| - Body fat percentage |  |  |  |  |
| **2. Flexibility** | | | | |
| - sit-and-reach test (cm) |  |  |  |  |
| **3. Strength** | | | | |
| - Handgrip strength (left) (kg) |  |  |  |  |
| - Handgrip strength (right) (kg) |  |  |  |  |
| - Leg strength (kg) |  |  |  |  |
| - Back strength (kg) |  |  |  |  |
| **4. Cardiovascular fitness and aerobic endurance** | | | | |
| - Heart rate per minute |  |  |  |  |
