# Supplementary material for: Associations of reallocating time between movement behaviours with adiposity and physical fitness among employees: a compositional data analysis
Source: BMC Public Health. 2025 May 20;25:1861. doi: 10.1186/s12889-025-23165-6 (PMC12090509; doi:10.1186/s12889-025-23165-6)
Supplement: Supplementary file 2 — Additional file 2. Sociodemographic and health behaviour questionnaire the questionnaire translated into English to collect data on participants’ personal information, sociodemographic characteristics, and health behaviours. [file 12889_2025_23165_MOESM2_ESM.docx]

**Sociodemographic and health behaviour survey**

**‘Development of Academic Tools and Evidence to Support Policy Design and Decision Making in Physical Activity Promotion to Reduce NCDs’ Project**

**Institute for Population and Social Research, Mahidol University**

**Respondent ID** **🞎🞎-🞎🞎-🞎🞎-🞎🞎🞎**

**(Date – Month – Interviewer no. – Respondent no.)**

**Accelerometer no. …………………………………………………**

**Personal information**

Full name ..................................................................................................................................................

Telephone ..................................................................................................................................................

Address ..................................................................................................................................................

..................................................................................................................................................

Workplace ..................................................................................................................................................

Occupation ..................................................................................................................................................

Position ..................................................................................................................................................

Recording Date ............................. Month ............................................. Year ..........................................

**Sociodemographic information**

1. Gender

1. Male 2. Female

1. Age ..................................... years
2. Household area

1. Urban 2. Rural

1. Marital status

1. Single 2. Married 3. Not married, but lived together

4. Widowed 5. Divorced/separated 9. Not identified

1. Highest education level

1. Below primary education 2. High school 3. Diploma

4. Bachelor degree 5. Higher than bachelor degree 9. Not identified

1. What type of work do you do?

1. Office work 2. Production work 3. Service work

4. Management work 5. Other work

1. What is your predominant posture at work?

1. Mostly sitting 2. Mostly standing 3. Mostly walking

4. Mostly using labour 5. Others

1. How much is your average income per month?

1. ≤ 10,000 baht 2. 10,001 – 20,000 baht 3. 20,001 – 30,000 baht

4. 30,001 – 40,000 baht 5. 40,001 – 50,000 baht 6. 50,001 – 100,000 baht

7. > 100,000 baht

**Health behaviours**

1. Do you currently smoke (including e-cigarette)?

1. Never smoked 2. Used to smoke 3. Currently smoke

1. Do you currently drink alcoholic beverages (e.g., liquor, beer, wine)?

1. Never drank 2. Drink once a month or less

3. Drink 2 – 4 times/month 4. Drink 2 – 3 times/week 5. Drink at least 4 time/week

1. On average each day, how much sweetened beverages (e.g., soft drinks, fruit juices, tea, coffee) do you drink?

1. Drink only water 2. Drink less than 1 bottle/box/can/glass

3. Drink 1 – 2 bottle/box/can/glass 4. Drink more than 2 bottle/box/can/glass

1. On average each day, how many ladles of fresh fruits and vegetables do you eat? (ladle = rice ladle)

1. More than 5 ladles 2. 4 – 5 ladles 3. 2 – 3 ladles

4. 1 ladle 5. Less than 1 ladle
