# Supplementary material for: Associations of reallocating time between movement behaviours with adiposity and physical fitness among employees: a compositional data analysis
Source: BMC Public Health. 2025 May 20;25:1861. doi: 10.1186/s12889-025-23165-6 (PMC12090509; doi:10.1186/s12889-025-23165-6)
Supplement: Supplementary file 3 — Additional file 3. Arithmetic means of adiposity markers and physical fitness by sample characteristics the detailed table of arithmetic means of adiposity markers and physical fitness by sample characteristics. [file 12889_2025_23165_MOESM3_ESM.docx]

**Arithmetic means of adiposity markers and physical fitness by sample characteristics**

| Variable | BMI  (kg/m2) | | Percent fat  (%) | | Waist circumference (cm) | | VO_2 max_  (mL/kg/min) | | Handgrip strength  (kg) | | Leg strength  (kg) | | Back strength  (kg) | | Flexibility  (cm) | |
| --- | --- | --- | --- | --- | --- | --- | --- | --- | --- | --- | --- | --- | --- | --- | --- | --- |
|  | Mean  (SD) | *p* | Mean  (SD) | *p* | Mean  (SD) | *p* | Mean  (SD) | *p* | Mean  (SD) | *p* | Mean  (SD) | *p* | Mean  (SD) | *p* | Mean  (SD) | *p* |
| **Sex** |  |  |  |  |  |  |  |  |  |  |  |  |  |  |  |  |
| - Female | **24.08**  **(4.67)** | **<0.001** | **33.49**  **(12.36)** | **<0.001** | **78.14**  **(11.57)** | **<0.001** | **37.00**  **(4.41)** | **<0.001** | **24.67**  **(4.37)** | **<0.001** | **60.90**  **(23.72)** | **<0.001** | **51.61**  **(15.79)** | **<0.001** | **7.06**  **(9.29)** | **0.001** |
| - Male | **26.15**  **(4.10)** |  | **23.41**  **(5.65)** |  | **90.21**  **(11.80)** |  | **45.68**  **(11.06)** |  | **39.58**  **(6.70)** |  | **115.05**  **(37.73)** |  | **93.33**  **(26.57)** |  | **3.70**  **(9.45)** |  |
| **Age** | 0.10 | 0.052 | **0.13** | **0.012** | 0.09 | 0.092 | **0.19** | **<0.001** | -0.02 | 0.736 | 0.04 | 0.500 | 0.01 | 0.892 | 0.08 | 0.110 |
| **Highest education level** |  |  |  |  |  |  |  |  |  |  |  |  |  |  |  |  |
| - Below bachelor degree | **26.79**  **(4.83)** | **0.029** | 27.42  (9.35) | 0.413 | **88.48**  **(16.51)** | **0.011** | 41.53  (9.38) | 0.509 | **37.72**  **(9.34)** | **<0.001** | **101.40**  **(43.43)** | **0.009** | **88.73**  **(34.37)** | **<0.001** | 5.81  (10.49) | 0.198 |
| - Bachelor degree | **24.52**  **(4.45)** |  | 30.21  (12.50) |  | **81.44**  **(11.99)** |  | 39.84  (8.83) |  | **29.58**  **(8.48)** |  | **78.89**  **(38.71)** |  | **65.43**  **(27.02)** |  | 6.30  (9.09) |  |
| - Higher than bachelor degree | **25.07**  **(4.74)** |  | 29.31  (7.28) |  | **83.93**  **(14.46)** |  | 40.66  (7.19) |  | **28.51**  **(8.94)** |  | **78.25**  **(37.43)** |  | **62.06**  **(27.53)** |  | 3.90  (10.49) |  |
| **Marital status** |  |  |  |  |  |  |  |  |  |  |  |  |  |  |  |  |
| - Never married | 24.69  (5.00) | 0.569 | 28.97  (8.17) | 0.149 | 82.15  (14.11) | 0.579 | 39.69  (8.75) | 0.280 | 29.99  (9.22) | 0.802 | 79.43  (37.75) | 0.492 | 66.61  (29.49) | 0.856 | **4.82**  **(9.40)** | **0.025** |
| - Currently or previously married | 24.97  (3.99) |  | 30.83  (14.54) |  | 82.90  (11.57) |  | 40.68  (8.42) |  | 30.22  (8.60) |  | 82.33  (41.24) |  | 67.16  (27.48) |  | **7.07**  **(9.47)** |  |
| **Monthly income** |  |  |  |  |  |  |  |  |  |  |  |  |  |  |  |  |
| - Very low (≤10,000 baht) | 27.82  (1.50) | 0.741 | 38.65  (2.05) | 0.365 | 88.12  (6.19) | 0.549 | 38.47  (4.18) | 0.505 | 25.05  (3.89) | 0.594 | 62.25  (36.42) | 0.802 | 40.00  (3.54) | 0.731 | 1.50  (16.26) | 0.720 |
| - Low (10,001 – 20,000 baht) | 24.55  (4.81) |  | 28.32  (7.64) |  | 81.42  (13.56) |  | 40.14  (8.32) |  | 31.02  (9.34) |  | 80.55  (40.11) |  | 67.22  (30.84) |  | 6.26  (8.81) |  |
| - Moderate (20,001 – 40,000 baht) | 24.75  (4.53) |  | 30.76  (15.41) |  | 82.17  (11.96) |  | 39.36  (7.82) |  | 29.49  (8.83) |  | 78.83  (38.85) |  | 66.59  (29.08) |  | 5.47  (9.46) |  |
| - High (40,001 – 100,000 baht) | 25.21  (4.48) |  | 30.11  (7.19) |  | 84.15  (14.38) |  | 41.47  (10.20) |  | 29.86  (8.64) |  | 84.69  (40.64) |  | 67.86  (25.50) |  | 6.33  (10.44) |  |
| - Very high (>100,000 baht) | 25.28  (2.86) |  | 28.47  (7.20) |  | 85.72  (10.31) |  | 41.23  (9.64) |  | 31.44  (8.94) |  | 83.37  (25.57) |  | 63.12  (13.76) |  | 2.50  (9.49) |  |
| **Predominant posture at work** |  |  |  |  |  |  |  |  |  |  |  |  |  |  |  |  |
| - Sitting | 24.91  (4.73) | 0.510 | 30.27  (12.87) | 0.119 | 82.84  (12.80) | 0.432 | 39.82  (8.78) | 0.256 | 29.88  (8.91) | 0.472 | 80.65  (39.14) | 0.952 | 65.72  (28.10) | 0.243 | 5.29  (9.63) | 0.074 |
| - Non-sitting | 24.58  (4.15) |  | 28.63  (6.77) |  | 81.60  (13.59) |  | 40.93  (8.14) |  | 30.64  (9.02) |  | 80.93  (40.00) |  | 69.74  (29.67) |  | 7.23  (9.00) |  |
| **Smoking** |  |  |  |  |  |  |  |  |  |  |  |  |  |  |  |  |
| - Never smoked | 24.61  (4.68) | 0.062 | **30.85**  **(12.02)** | **<0.001** | **81.28**  **(12.86)** | **<0.001** | **39.44**  **(8.28)** | **0.001** | **28.44**  **(8.24)** | **<0.001** | **74.32**  **(36.67)** | **<0.001** | **61.47**  **(25.58)** | **<0.001** | 6.12  (9.51) | 0.331 |
| - Former smoker | 25.19  (3.48) |  | **24.16**  **(4.99)** |  | **88.67**  **(10.92)** |  | **44.53**  **(10.02)** |  | **37.08**  **(7.61)** |  | **109.00**  **(39.58)** |  | **90.00**  **(29.16)** |  | 3.05  (9.91) |  |
| - Current smoker | 26.46  (3.89) |  | **24.73**  **(6.49)** |  | **89.24**  **(12.53)** |  | **43.41**  **(9.05)** |  | **39.58**  **(7.28)** |  | **115.97**  **(36.00)** |  | **97.23**  **(26.73)** |  | 5.11  (8.90) |  |
| **Alcohol consumption** |  |  |  |  |  |  |  |  |  |  |  |  |  |  |  |  |
| - Non-drinker | 24.49  (4.52) | 0.092 | **31.77**  **(14.61)** | **0.009** | **81.15**  **(12.69)** | **0.049** | **38.27**  **(6.53)** | **<0.001** | **27.41**  **(7.38)** | **<0.001** | **73.45**  **(35.70)** | **0.002** | **60.17**  **(24.75)** | **<0.001** | 6.10  (9.70) | 0.779 |
| - Light to moderate drinker | 24.91  (4.72) |  | **28.48**  **(7.69)** |  | **83.10**  **(12.87)** |  | **41.44**  **(9.73)** |  | **31.93**  **(9.69)** |  | **85.09**  **(42.26)** |  | **70.54**  **(30.63)** |  | 5.76  (9.22) |  |
| - Heavy drinker | 26.64  (3.42) |  | **26.24**  **(6.90)** |  | **87.73**  **(14.99)** |  | **43.95**  **(9.96)** |  | **35.13**  **(7.81)** |  | **97.10**  **(33.84)** |  | **85.08**  **(26.20)** |  | 4.67  (10.06) |  |
| **Daily sweetened beverage intake** | | | | | | | | | | | | | | | | |
| - None | **25.68**  **(4.24)** | **0.041** | 29.68  (7.76) | 0.830 | **84.91**  **(12.34)** | **0.032** | 40.11  (8.52) | 0.984 | 31.23  (10.33) | 0.243 | **92.80**  **(42.17)** | **0.023** | **75.07**  **(30.31)** | **0.005** | 5.43  (9.30) | 0.445 |
| - Light | **24.45**  **(4.76)** |  | 30.63  (16.96) |  | **80.91**  **(14.28)** |  | 40.14  (8.04) |  | 29.55  (8.43) |  | **80.44**  **(38.27)** |  | **67.50**  **(27.55)** |  | 6.96  (8.78) |  |
| - Moderate | **24.55**  **(4.35)** |  | 29.33  (7.53) |  | **82.06**  **(12.16)** |  | 40.23  (9.17) |  | 29.66  (8.81) |  | **75.39**  **(37.97)** |  | **62.07**  **(27.02)** |  | 5.37  (9.71) |  |
| - Heavy | **27.31**  **(5.87)** |  | 30.13  (9.82) |  | **89.58**  **(12.06)** |  | 39.33  (6.98) |  | 33.54  (7.58) |  | **88.75**  **(43.27)** |  | **78.72**  **(35.97)** |  | 4.06  (12.29) |  |
| **Daily fruit and vegetable intake** | | | | | | | | | | | | | | | | |
| - Sufficient | 24.23  (4.12) | 0.348 | 27.47  (7.30) | 0.055 | 82.47  (11.10) | 0.983 | 39.80  (6.84) | 0.752 | 32.08  (9.65) | 0.178 | 85.01  (38.65) | 0.458 | 72.46  (31.97) | 0.246 | 6.26  (8.88) | 0.761 |
| - Insufficient | 24.90  (4.62) |  | 30.12  (11.88) |  | 82.52  (13.23) |  | 40.18  (8.79) |  | 29.83  (8.84) |  | 80.04  (39.51) |  | 66.09  (28.14) |  | 5.79  (9.55) |  |
| **Blood pressure** |  |  |  |  |  |  |  |  |  |  |  |  |  |  |  |  |
| - Normal | **24.24**  **(4.06)** | **<0.001** | 29.57  (11.88) | 0.281 | **81.15**  **(11.71)** | **<0.001** | 39.76  (8.37) | 0.057 | **29.39**  **(8.88)** | **<0.001** | 78.98  (39.23) | 0.062 | **65.40**  **(28.35)** | **0.039** | 5.85  (9.41) | 0.910 |
| - High | **27.96**  **(5.38)** |  | 31.05  (8.57) |  | **89.88**  **(16.68)** |  | 42.48  (9.54) |  | **34.00**  **(8.41)** |  | 90.26  (39.64) |  | **74.60**  **(29.22)** |  | 6.02  (9.88) |  |

Notes: BMI = body mass index; VO_2_max = maximum oxygen consumption; SD = standard deviation; *p* = p-value; Significance was indicated in bold.
